# Metasurface-based Fourier ptychographic microscopy

**DOI:** 10.1515/nanoph-2025-0416

**Published:** 2025-11-25

**Authors:** Cheng Hung Chu, Hao-Pin Chiu, Cheng Yu, Yuan-Chung Cheng, Ching-En Lin, Sunil Vyas, Yuan Luo

**Affiliations:** Institute of Medical Device and Imaging, 33561 National Taiwan University, Taipei 10051, Taiwan; YongLin Institute of Health, National Taiwan University, Taipei 10672, Taiwan; Department of Medicine, National Taiwan University, Taipei 10051, Taiwan; Department of Physics, National Taiwan University, Taipei 10617, Taiwan; Program for Precision Health and Intelligent Medicine, National Taiwan University, Taipei 106319, Taiwan; Institute of Biomedical Engineering, National Taiwan University, Taipei 10051, Taiwan

**Keywords:** metalens, quantitative phase imaging, residual convolutional neural network, Fourier ptychographic microscopy

## Abstract

Meta-optics have opened new possibilities for portable, high-performance microscopy, offering ultrathin and highly customizable wavefront control in scenarios where bulky optics limit adoption. Here, we use this capability to overcome the long-standing challenges of Fourier ptychography (FP), a powerful computational technique for wide-field, high-resolution quantitative phase imaging that traditionally depends on large optical elements and extensive angle scanning. Our compact meta-FP platform combines a 4-f metalens system for imaging miniaturization with a programmable thin-film transistor (TFT) panel to provide stable, angle-diverse plane-wave illumination without mechanical movement. To further accelerate imaging, we introduce a residual convolutional neural network (RCNN) model trained via transfer learning on conventional FP datasets, which allows for single-shot inference of high-resolution phase from low-resolution inputs. Experimental validations demonstrate nearly twofold resolution improvement (7.81 µm–3.91 µm), accurate quantitative phase recovery on phase standards with errors below 10 %, and dry-mass estimation of H1975 cells with an average deviation of approximately 12 %, while the best-performing regions exhibit deviations below 0.5 %. This integration of metasurface optics and artificial intelligence-driven reconstruction provides a promising pathway for fast and compact FP microscopy with applications in live-cell imaging, microfluidic monitoring, and point-of-care diagnostics.

## 1 Introduction

Fourier ptychographic (FP) microscopy has emerged as a powerful computational imaging technique, enabling quantitative phase imaging (QPI) with both high resolution and a wide field of view (FOV) [1], [2], [3], [4], [5], [6], [7], [8], [9], [10], [11], [12], [13], [14], [15]. By synthesizing a large effective numerical aperture (NA) through angularly multiplexed low-resolution intensity measurements, FP microscopy can simultaneously retrieve intensity and phase information, which is essential for the structural and functional analysis of transparent biological specimens and material characterization. Compared to conventional QPI techniques such as digital holography [16], [17], [18], FP microscopy does not require interferometric configurations, making it more robust against mechanical instabilities and environmental perturbations. Despite these advantages, traditional FP systems face two major challenges. First, the use of bulky optical components, including large objectives and relay optics, results in complex and heavy systems that are difficult to integrate into portable or point-of-care devices. Second, FP microscopy inherently requires a large number of images under varying illumination angles to achieve the desired synthetic NA, leading to prolonged data acquisition times and limiting its applicability to dynamic biological samples.

Recently, metasurfaces have emerged as a breakthrough in nanophotonics, fundamentally reshaping optical design [19], [20], [21], [22], [23]. Unlike bulky refractive optical elements, metasurfaces leverage subwavelength nanostructures to manipulate amplitude, phase, and polarization of light with unprecedented precision within an ultrathin, planar form factor. This high flexibility enables precise and versatile control in wavefront engineering, circumventing the complexity of traditional optics. Metalenses, a prominent subset of metasurfaces, have demonstrated remarkable success in compact imaging systems, including endoscopic microscopy, augmented reality (AR) displays, etc. [24], [25], [26], [27], [28], [29], [30], [31], [32], [33], [34], [35], [36]. By replacing multi-element lens assemblies with a single planar component, metalenses significantly reduce system size while delivering the desired functionality, making them an ideal candidate for integration into advanced microscopy platforms. In recent years, metasurfaces have attracted significant attention in various computational imaging modalities, including hyperspectral, polarization-resolved, and three-dimensional (3D) imaging [37], [38], [39], [40], [41], [42]. In addition, metasurfaces have also been directly employed in QPI studies [43]. For instance, metasurface-based digital holographic microscopy (DHM) has been demonstrated by integrating phase-gradient or beam-splitting metasurfaces to enable compact interferometric configurations, allowing single-shot phase retrieval [44]. Transport-of-intensity equation (TIE) based QPI has further benefited from metasurface elements that tailor defocus or generate multi-focal fields for simultaneous acquisition of phase diversity [45], [46], [47]. These works demonstrate that metasurfaces provide significant benefits, including system miniaturization, wavefront shaping flexibility, and improved optical performance. While metasurfaces have been widely adopted in various imaging systems, their integration into the core optical architecture of Fourier ptychography remains an open and largely untapped area.

In this work, we address this gap by introducing a metasurface-based FP (meta-FP) microscopy system featuring a compact meta-microscope for quantitative phase imaging. This design achieves significant miniaturization of the FP system while retaining the capacity for high-resolution quantitative phase imaging. Furthermore, to address the acquisition burden of FP microscopy, and the reduced NA and degraded image quality due to physical constraints, we introduce a residual convolutional neural network (RCNN) deep learning model trained on high-resolution data from a home-built FP microscope equipped with a commercial high-NA objective, using a transfer learning method [48]. The well-trained model is applied to single-shot low-resolution images acquired under central illumination by the meta-FP system, and directly translates them into high-resolution outputs, eliminating the need for multi-angle images acquisitions.

## 2 Results

**Figure 1:**
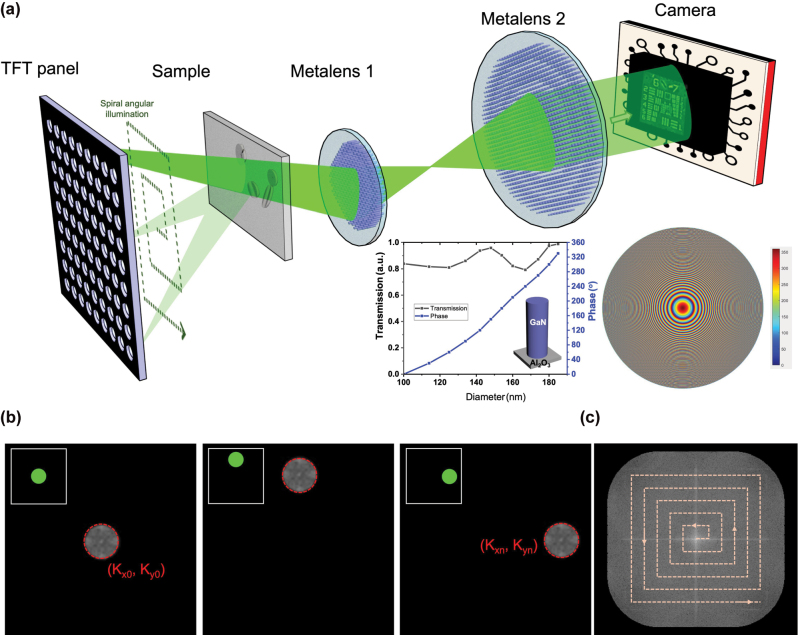
Experimental setup and illumination scheme of the proposed meta-FP microscopy system. (a) Schematic diagram of the system configuration. The inset plots illustrate the simulated phase shift and transmission efficiency of GaN-based cylindrical nanopillars as a function of diameter (left), and the central phase profile of one metalens (right). (b) Fourier spectrum converted from low-resolution images acquired under three selected illumination angles (c) Fourier domain patch updating process, where each low-resolution image contributes a local patch extracted from its corresponding Fourier spectrum and is merged to construct the high-resolution Fourier domain.

The optical setup of the proposed meta-FP microscopy system with dynamic illumination control is shown in Figure 1(a). A programmable thin-film transistor (TFT) panel (Seeed, 2.8” TFT Touch Shield V2.0), illuminated by a laser-driven light source (LDLS) which is filtered at a central wavelength of 532 nm and coupled through a collimation module to produce a uniform plane-wave illumination across the panel surface, serves as the core of the angularly tunable illumination module by selectively activating pinhole-like transmission through its addressable pixels. The TFT panel consists of a 320 × 240 grid of individually addressable pixels, each with a pixel size of 180 μm. Because the illumination is collimated and the propagation distance from the TFT panel to the sample (∼40 mm) is much larger than the pixel size, the optical field incident on the sample can be regarded as a plane wave within the illumination area. A 9 × 9 array of central pixels (corresponding to 81 illumination angles) is employed to provide sufficient angular diversity for stable FP phase retrieval. It provides approximately 40 % overlap between neighboring frequency patches, which is known to support robust convergence [49]. Compared to sparser configurations such as 5 × 5, as shown in Figure S1, the 9 × 9 grid significantly improves reconstruction quality, while denser sampling schemes offer only marginal gains at the cost of longer acquisition and computational time. The angle-varying illumination is dynamically controlled by sequentially switching the TFT pixels on and off in a spiral pattern, enabling programmable directional lighting without mechanical scanning. The dataset acquisition time for each raw FP image is approximately 0.75 s, resulting in a total acquisition time of about 60 s for the complete set of 81 illumination angles. The TFT panel offers pixel-level programmable illumination similar to LED arrays, but with several important advantages [50]. It has higher pixel density, which enables the generation of finer spatial patterns and greater degrees of freedom in illumination control. While conventional LED emitters typically have millimeter-scale dimensions, the TFT panel features sub-millimeter (hundreds of micrometers) pixels, which enhances spatial coherence and thereby improves the resolution of FP reconstruction [10]. It also delivers excellent illumination uniformity across large areas, supported by well-engineered backlight diffusion. In addition, the mature manufacturing infrastructure helps reduce system cost, and the thin, compact form factor makes integration easier. They also allow the use of high-power external light sources, making it easier to increase the photon budget without being constrained by the physical size of individual LEDs. Furthermore, the illumination intensity remains stable when switching patterns, avoiding the pixel-to-pixel variation and temporal drift that are common in LED arrays. Together, these features position TFT panels as a highly efficient and economical solution for programmable light control, especially in applications that require complex, high-resolution, and rapidly switchable illumination patterns. Moreover, their planar design is well-suited for miniaturized, modular, and embedded optical systems, expanding their potential use in advanced microscopy and integrated photonics.

Following the sample plane, light propagates through a compact 4-f imaging system composed of two metalenses, metalens 1 with a focal length (FL) of 13 mm and a diameter of 2.6 mm, and metalens 2 with an FL of 50 mm and a diameter of 5.2 mm, resulting in about 4× magnification with pupil matching between elements to maintain optimal light throughput. These metalenses are designed using CST Studio Suite and fabricated using electron beam lithography, as described in the Methods section. The final images are captured by a CMOS sensor with a pixel size of 1.75 μm × 1.75 μm, positioned at the imaging plane of the second metalens. As a result, the end-to-end optical path from the TFT panel to the camera sensor is about 17 cm.

The FP reconstruction process is initiated by acquiring multiple low-resolution intensity images under varying illumination angles. Each angularly modulated illumination results in a lateral shift of the object’s Fourier spectrum. Figure 1(b) illustrates the corresponding shifted spectral regions for three selected illumination angles, each centered at a distinct illumination wavevector (*k*_*x*_, *k*_*y*_). The algorithm initializes the reconstruction by upsampling the Fourier transform of the on-axis (normal incidence) image to generate a preliminary high-resolution Fourier spectrum. For each additional illumination angle, a circular region of the spectrum, corresponding to the shifted coherent transfer function, is extracted and subjected to inverse Fourier transform to obtain a complex field estimate in the spatial domain. The amplitude of this estimate is then replaced with the square root of the measured intensity, while its phase is retained. The updated complex field is subsequently transformed back to the Fourier domain, yielding a new spectral patch. As shown in Figure 1(c), this patchwise update is inserted into the corresponding location in the Fourier spectrum. This process is repeated for all illumination angles, with each iteration refining the Fourier spectrum by updating only the regions corresponding to each illumination, leaving the remainder unchanged. Multiple cycles of this update process are performed, during which the estimates of both amplitude and phase are progressively improved. Empirically, convergence is achieved after five iterations, beyond which no substantial improvements are observed. After acquisition, the high-resolution image is reconstructed using an iterative Fourier ptychographic algorithm, which completes within 3 s. The final high-resolution complex image is obtained by applying an inverse Fourier transform to the fully refined spectrum, effectively synthesizing a numerical aperture larger than the physical limit of the metalens. Details of the FP reconstruction algorithm are provided in Supporting Information, Section 2.

**Figure 2:**
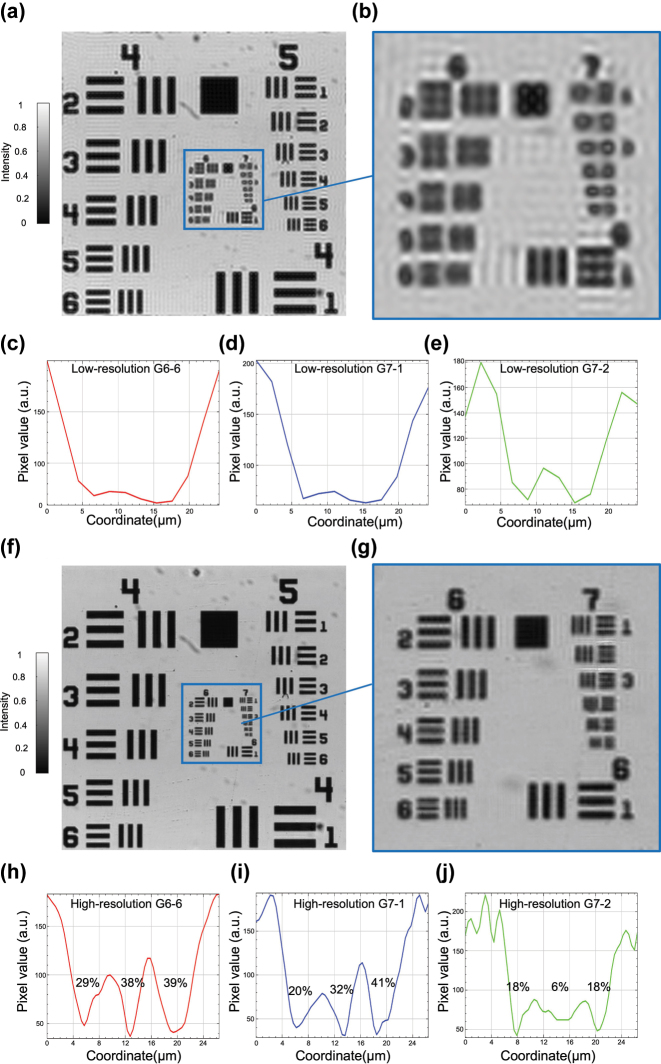
Resolution enhancement of the metasurface-based FP microscopy system demonstrated on a USAF resolution target. (a) Low-resolution image acquired under central illumination. (b) Zoomed-in view of the central region containing Group 6 and Group 7 (denoted as G6 and G7 hereafter). (c)–(e) Cross-sectional intensity profiles of Elements 6, 1, and 2 from G6 and G7 (noted as G6-6, G7-1, and G7-2, respectively) extracted from the low-resolution image, showing weak contrast and blurred features. (f) High-resolution image reconstructed by FP. (g) Zoomed-in view of the corresponding region from (f) with significantly enhanced resolution and contrast. (h)–(j) Cross-sectional intensity profiles from the high-resolution image corresponding to G6-6, G7-1, and G7-2, exhibiting clear intensity oscillations and improved contrast.

To evaluate the resolution enhancement capability of our metasurface-based FP microscopy system, we image a 1951 USAF resolution target. The low-resolution image acquired using illumination from the central pixel of the TFT reveals significant blurring and loss of fine structural features, attributable to the limited NA of the optical system, as illustrated in Figure 2(a). Figure 2(b) shows a magnified view of the central region, encompassing Groups 6 and 7 of the resolution target, where high-frequency spatial details are not resolvable. This limitation is further confirmed by the intensity profiles of several representative elements, including G6-6, G7-1, and G7-2, as shown in Figure 2(c)–(e). All profiles exhibit smooth intensity variations with poor contrast and lack of discernible peaks, underscoring the inadequate resolution of the low-NA system.

Following FP reconstruction, the synthesized high-resolution image demonstrates substantial improvement in visual clarity and feature definition, as shown in Figure 2(f). The reconstructed image incorporates information from multiple angular illuminations, effectively extending the system’s synthetic NA and recovering previously inaccessible high-frequency content. A zoomed-in view of the same region is presented in Figure 2(g), highlighting the restored visibility of fine features within G6 and G7. The intensity profiles of the reconstructed image are displayed in Figure 2(h)–(j), revealing improved contrast. To quantitatively evaluate this enhancement, we use the Michelson contrast, defined as

$$C=\frac{I_{\max}-I_{\min}}{I_{\max}+I_{\min}},$$

where *I*_max_ and *I*_min_ denote the peak and valley intensities within a periodic pattern. Using this metric, the contrast values are measured as 29–39 % for G6-6, 20–41 % for G7-1, and 6–18 % for G7-2, confirming the effective recovery of high-frequency information and improved feature visibility. Additionally, a comparison of the smallest resolvable features further supports the resolution enhancement. In the low-resolution image, the smallest identifiable element is G6-1, corresponding to a resolution limit of 7.81 µm. After FP reconstruction, G7-1, corresponding to 3.91 µm, becomes clearly distinguishable, indicating a nearly twofold improvement in resolution.

**Figure 3:**
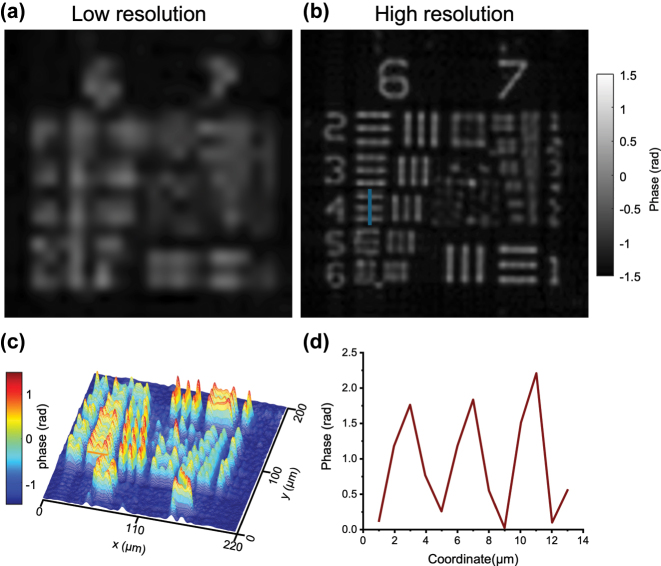
Phase imaging results of a USAF phase target using the proposed meta-FP microscopy system. (a) Low-resolution phase image reconstructed from a single central illumination. (b) High-resolution phase image reconstructed by FP. (c) 3D visualization of the retrieved phase map of (b). (d) Retrieved phase profile along G6-4, where the minimum phase level is shifted to zero for direct comparison with the theoretical phase value.

Figure 3 shows the quantitative phase imaging performance of the meta-FP system using a USAF phase resolution target. The low-resolution phase image reconstructed from central illumination (Figure 3a) exhibits significant blurring and loss of fine structure due to the limited NA. In contrast, the FP-reconstructed high-resolution image (Figure 3b) reveals well-defined phase features corresponding to G6 and G7 of the target. To visualize the retrieved phase distribution, Figure 3(c) presents a 3D rendering of the reconstructed phase across the central region. Figure 3(d) shows a quantitative phase profile extracted along G6-4. Based on a known thickness (*D* = 350 nm) and refractive index difference (Δ*n* = 1.52), the expected phase shift is 2.15 radians at *λ* = 532 nm, while the reconstructed phase difference reaches approximately 1.95 radians, corresponding to a relative error of 9.4 %. This slight underestimation of the measured phase is associated with the central depression observed in the reconstructed phase map, both originating from the incomplete recovery of the lowest spatial frequency components that suppress large-scale phase variations. This phenomenon originates from the non-uniform phase transfer characteristic of conventional FP, where low-frequency phase variations produce nearly constant intensity images and thus cannot be accurately encoded or reconstructed [51].

**Figure 4:**
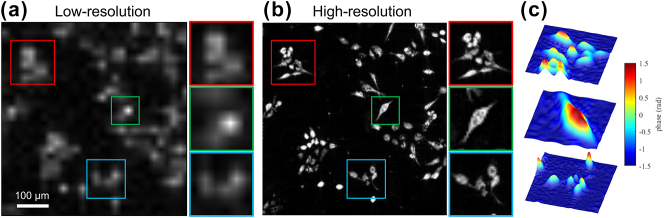
Quantitative phase imaging results of fixed H1975 cells immersed in phosphate-buffered saline (PBS) on a glass slide, using the proposed meta-FP microscopy system. (a) Low-resolution phase image acquired under single-angle illumination, with magnified views of selected ROIs (red, green, and blue boxes). (b) High-resolution phase image reconstructed by the FP algorithm. (c) 3D renderings of the retrieved phase distributions corresponding to the boxed regions in (b), highlighting spatial phase variations within individual cells.

Figure 4 presents the quantitative phase imaging results of fixed H1975 cells using our meta-FP microscopy system. H1975 is a human non-small cell lung cancer (NSCLC) adenocarcinoma cell line characterized by concurrent EGFR L858R and T790M mutations, and is widely employed in studies of targeted therapy efficacy and mechanisms of drug resistance in lung cancer. The low-resolution phase image with three regions of interest (ROIs) in Figure 4(a), acquired under single-angle center illumination, exhibits limited spatial resolution and low contrast, resulting in blurred cellular structures and unresolved subcellular features. In contrast, the high-resolution phase image reconstructed by the FP algorithm, shown in Figure 4(b), reveals clear morphological features, including well-defined cell boundaries and internal structures that are indistinct in the low-resolution image. Figure 4(c) displays 3D renderings of the recovered phase distributions from the regions marked in Figure 4(b). These localized phase maps highlight spatially-resolved optical path length differences and morphological contrast across individual cells. Together, these results validate the effectiveness of meta-FP system in reconstructing high-resolution phase maps for label-free biological imaging.

**Figure 5:**
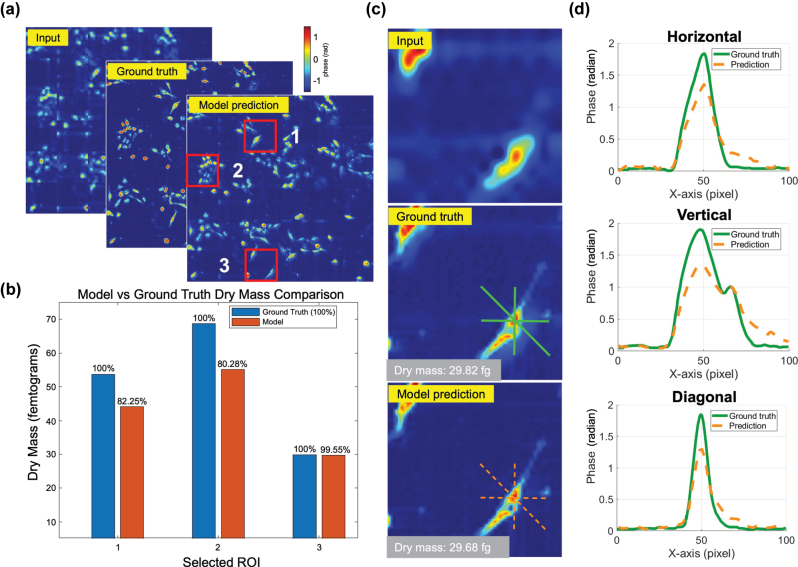
Quantitative evaluation of the RCNN-FP model applied to H1975 cell phase reconstruction. (a) Comparison of low-resolution input, ground truth, and model prediction. (b) Dry mass estimation accuracy across three selected ROIs. (c) Detailed phase image comparison for ROIs. (d) Cross-sectional phase profiles along horizontal, vertical, and diagonal directions.

We develop an RCNN model to accelerate FP image reconstruction by predicting high-resolution images from low-resolution inputs. The model architecture is detailed in Figure S3. The training is conducted using a diverse dataset comprising various biological specimens imaged with a conventional FP microscope, as illustrated in Figures S4 and S5. The network is optimized to learn the mapping between low- and high-resolution phase image pairs. After training, the model is applied to previously unseen H1975 cell images captured by the meta-FP microscope, which served as the testing dataset. A summary of the evaluation results is provided in Figure 5. As shown in Figure 5(a), the predicted phase images exhibit strong visual agreement with the multi-angle FP ground truth. With the trained RCNN-FP model, the high-resolution phase can be reconstructed from a single image in less than 1 s, achieving more than an order-of-magnitude reduction in total imaging time.

To quantitatively assess model performance, we computed the dry mass within three selected ROIs [52], as annotated in Figure 5(a). The definition and calculation of dry mass are provided in Supporting Information, Section 5. As shown in Figure 5(b), the predicted dry mass values deviate from the ground truth by an average of about 12 %, confirming the model’s reliability for quantitative phase analysis. A comparison is presented in Figure 5(c), which shows side-by-side predicted and ground truth phase maps for ROIs. The predicted and ground truth dry mass values are 29.68 and 29.82 fg (fg), respectively, with an error of less than 0.5 %. To evaluate local structural fidelity, phase profiles along horizontal, vertical, and diagonal cross-sections of the same cell are plotted in Figure 5(d), demonstrating excellent agreement between prediction and ground truth across all directions. Importantly, the model achieved high-fidelity reconstruction without the need for retraining or fine-tuning on the meta-FP dataset, highlighting the effectiveness of the transfer learning strategy. This framework enables rapid and accurate inference from single-shot images, substantially reducing the number of required acquisitions and total imaging time, a critical advancement toward practical, real-time FP microscopy.

## 3 Conclusion and outlook

We have developed a compact FP microscopy system that combines meta-optics with deep learning-driven image reconstruction for high-resolution quantitative phase imaging. The integration of a 4-f metalens configuration enables substantial miniaturization while preserving optical performance, and the TFT illumination panel provides programmable, stable angular control without the need for mechanical scanning. The overall optical path from the illumination panel to the image sensor is on the order of only a few tens of centimeters, which is several-fold shorter than conventional FP microscopes that rely on bulky objectives and tube-lens relays extending to over half a meter. This compact form factor underscores the advantage of metasurface integration for realizing portable computational imaging platforms. Together with the RCNN reconstruction framework, the system achieves single-shot, quantitative phase recovery with low error, significantly reducing the acquisition burden and enabling rapid imaging. Experimental validations on resolution targets and H1975 cells confirm the system’s ability to recover fine structural and biophysical information, including accurate dry mass estimation, highlighting its potential for live-cell imaging, point-of-care diagnostics, and microfluidic monitoring. These characteristics highlight the current strengths of our system and suggest potential directions for continued advancement in metalens design, active modulation, and deep reconstruction technologies.

While this work focuses on validating the feasibility of meta-FP microscopy, further improvements are possible. Looking ahead, further gains could be realized primarily via the metalens: dispersion-engineered achromatic designs in the visible to support broadband or multi-wavelength FP with consistent phase transfer, and aberration-controlled, large-FOV metalens doublets (e.g., monolithic double-sided or multilayer stacks) to preserve resolution at high field angles while maintaining an ultra-slim form factor [53], [54]. Additionally, tunable/adaptive metalenses could enable rapid focal sweep and *in-situ* aberration compensation, and folded metasurface layouts may provide long optical paths within millimeter-scale packages suitable for tight CMOS integration [55], [56]. On the FP side, optional upgrades include embedded pupil/aberration recovery (EPRY), illumination-angle self-calibration, multiplexed/structured (including polarization-coded) illumination to reduce frame count, and physics-informed or plug-and-play deep reconstructions, each of which has been shown to improve effective synthetic NA or robustness without adding substantial hardware complexity [7], [57], [58], [59]. Collectively, these directions not only consolidate the current technical advantages of our platform but also pave the way for broader and more versatile applications in quantitative phase imaging.

## 4 Methods

### 4.1 The design and fabrication of metalens

The metasurface was designed using the commercial electromagnetic simulation software CST Studio Suite (Microwave Studio), employing full-wave finite integration simulations. The meta-atoms consist of cylindrical GaN nanopillars arranged in a square lattice on a double-polished sapphire substrate. To approximate an infinitely periodic structure, periodic boundary conditions were applied in the *x*-*y* plane, while open boundaries were used along the *z*-axis to allow wave propagation. Simulations were conducted under normal incidence of a plane wave of wavelength of 532 nm, with polarization along either the *x*- or *y*-axis to confirm polarization-independent behavior. To achieve full 0-2π phase coverage with high transmittance, the nanopillar diameter was swept across a range of 100 nm–185 nm, while the height and period were set at 850 nm and 260 nm, respectively. The mapping between nanopillar diameter and corresponding phase shift *φ* was extracted and used to generate a metalens phase profile based on a hyperbolic phase function:

$$\varphi \left(r,\lambda \right)=-\frac{2\pi }{\lambda }\left(\sqrt{r^{2}+f^{2}}-f\right),$$

where *r* is the radial position, *λ* is the working wavelength, and *f* is the focal length. Because the lens phase profile is implemented through discrete unit cells, a phase quantization error of 0.17 rad, calculated as the root mean square error between the theoretical and simulated phase distributions, is introduced. The focusing behavior of the metalens was evaluated in Supporting Information, Section 6. As shown in Figure S7, the 850 nm-thick GaN metalenses were fabricated on a c-axis sapphire wafer. A 200 nm-thick SiO_2_ layer was deposited via electron-beam evaporation to serve as a hard mask. PMMA A4 was then spin-coated and patterned using electron beam lithography (EBL, Elionix ELS-HS50) at 50 kV. After exposure, the resist was developed using a MIBK:IPA (1:3) solution, followed by rinsing in IPA. A 40-nm-thick chromium layer was deposited onto the patterned sample by electron-beam evaporation and used as a hard mask. The lift-off process was performed using acetone to remove the residual resist. The nanostructure pattern was transferred from Cr to SiO_2_ using inductively coupled plasma reactive ion etching (ICP-RIE) with CF_4_ gas. The remaining Cr mask was removed using chromium etchant. A second ICP-RIE step, employing a Cl_2_-based gas mixture, was used to etch through the GaN layer. Finally, the residual SiO_2_ was removed by a buffered oxide etch (BOE), leaving behind only the GaN nanopillars on the sapphire substrate, thus forming the designed metasurface.

### 4.2 RCNN model implementation

A schematic of the residual convolutional neural network (RCNN) used for FP reconstruction is shown in Figure S3. The model adopts a U-net-inspired encoder-decoder structure, consisting of four residual downsampling blocks and four residual upsampling blocks. In the encoder path, the spatial resolution is reduced from 256 × 256 to 16 × 16 pixels while the number of feature channels increases progressively from 16 to 256. Each downsampling block is composed of two 3 × 3 convolutional layers with a stride of 1, followed by max pooling and a residual connection to ensure efficient gradient propagation. The decoder mirrors this structure, gradually restoring the spatial resolution to 256 × 256 pixels using upsampling layers and residual shortcut connections. Skip connections are included between encoder and decoder layers of the same resolution to preserve low-level spatial features. All convolutional layers use leaky ReLU activation (slope = 0.5), except for the final output layer, which uses a 1 × 1 convolution followed by a tanh activation function to produce the final high-resolution image. Dropout and batch normalization are applied throughout the network to enhance training stability.

The model was trained using the Adam (Adaptive moment estimation) optimizer, an adaptive learning rate algorithm. The initial learning rate was set to 1 × 10^−3^, and a learning rate scheduler was used to adjust it during training according to the equation:

$$L_{rate}=L_{rate\text{\_}initial}\times {drop}^{\frac{1+epoch}{epochs_{drop}}},$$

where drop is the decay factor (set to 1.0 in this work) and epochs_drop_ = 500 represents the decay interval. The model was trained for 5,000 epochs with a batch size of 14, using the mean absolute error (MAE) as the loss function. A total of 9,348 paired images (256 × 256 pixels) were collected from five types of biological cell and tissue samples to ensure data diversity. These were split into 6,486 training, 1,080 validation, and 1,782 testing images. The model was implemented using TensorFlow (v2.4.0) and trained on a workstation equipped with dual Intel Xeon Silver 4210R CPUs (2.4 GHz), 256 GB RAM, and two NVIDIA RTX A5000 GPUs (24 GB each). Training is completed in approximately 18.5 h with DNN acceleration, which was over 80× faster than CPU-only training.

## Supplementary Material

Supplementary Material Details
