# Supplementary material for: Metasurface-based Fourier ptychographic microscopy
Source: Nanophotonics. 2025 Nov 25;14(25):4595–605. doi: 10.1515/nanoph-2025-0416 (PMC12714035; doi:10.1515/nanoph-2025-0416)
Supplement: Supplementary file 1 — Supplementary Material Details [file j_nanoph-2025-0416_suppl_001.docx]

**Metasurface-Based Fourier Ptychographic Microscopy**

Cheng Hung Chu^1^, Hao-Pin Chiu^2^, Cheng Yu^3^, Yuan-Chung Cheng^2^, Ching-En Lin^4^, Sunil Vyas^2^, and Yuan Luo^1,2,5,6*^

^1^YongLin Institute of Health, National Taiwan University, Taipei 10672, Taiwan

^2^Institute of Medical Device and Imaging, National Taiwan University, Taipei 10051, Taiwan

^3^Department of Medicine, National Taiwan University, Taipei 10051, Taiwan

^4^Department of Physics, National Taiwan University, Taipei, 106, Taiwan

^5^Program for Precision Health and Intelligent Medicine, National Taiwan University, Taipei, 106319, Taiwan

^6^Institute of Biomedical Engineering, National Taiwan University, Taipei 10051, Taiwan

Section 1: Effect of the number of illumination angles on FP reconstruction quality


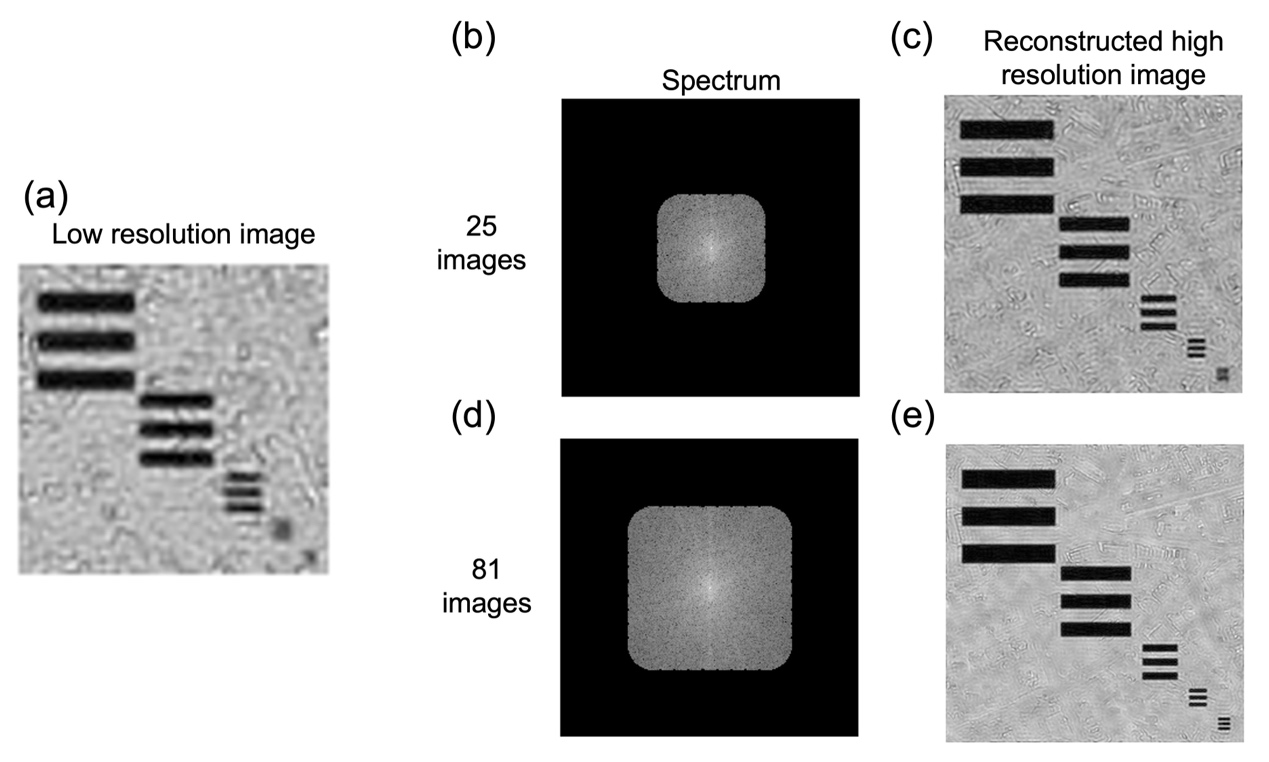


Figure S1. (a) Low-resolution image acquired under central illumination. (b) Simulated Fourier spectrum coverage obtained using 25 illumination angles. (c) Reconstructed high-resolution image from 25-angle data. (d) Fourier spectrum coverage with 81 illumination angles. (e) Reconstructed image with 81-angle data, showing improved spatial frequency coverage and resolution compared to (c).

To evaluate the effect of illumination angle density on FP reconstruction quality, we simulated two scenarios using 25 and 81 angular illuminations, respectively. As shown in Fig. S1(a), the input low-resolution image acquired under central illumination suffers from limited resolution due to the narrow angular coverage. Figures S1(b) and S1(d) present the corresponding synthetic Fourier spectrum coverage achieved with 25 and 81 illuminations. With fewer angles (Fig. S1(b)), the spectral coverage is limited, leading to insufficient sampling in high-frequency regions. This results in the reconstructed image (Fig. S1(c)) exhibiting noticeable blur and reduced detail fidelity. In contrast, using 81 illumination angles (Fig. S1(d)) significantly expands the spectral coverage, enabling the recovery of higher spatial frequencies. The corresponding reconstruction (Fig. S1(e)) demonstrates markedly enhanced resolution and improved feature clarity. These results highlight the importance of adequate angular sampling in FP and validate the choice of 81 angles in our meta-FP system to ensure stable and high-quality reconstructions.

Section 2: FP reconstruction


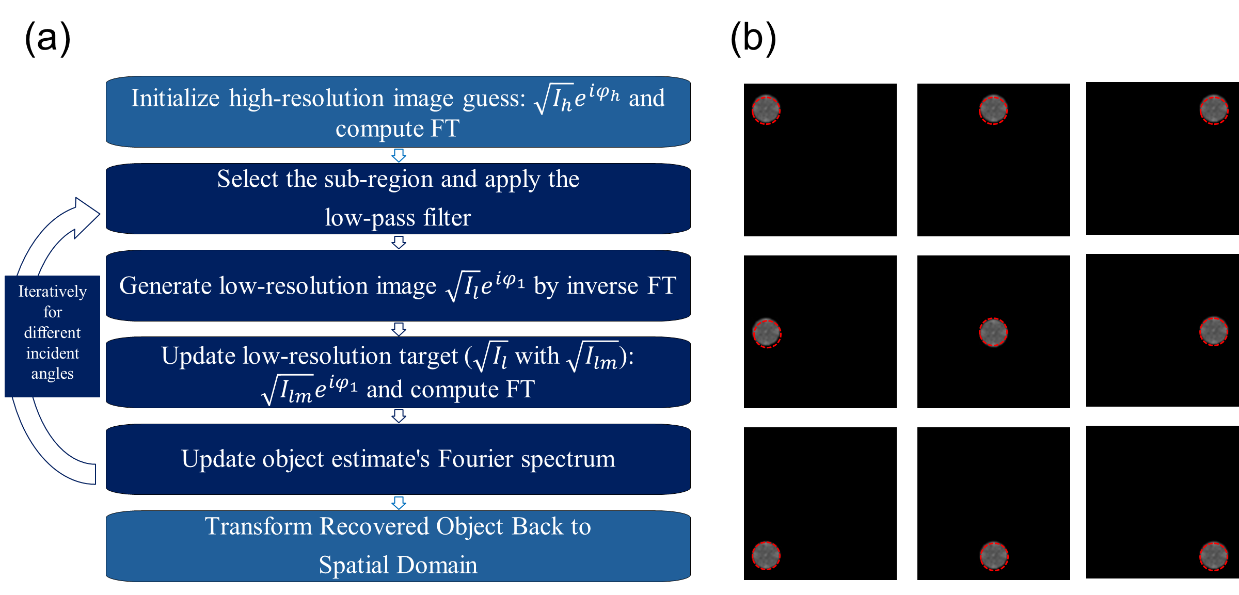


Figure S2. (a) Detailed workflow of the FP reconstruction process. (b) Low-resolution intensity images acquired under different illuminations.

Below, we provide a brief theoretical derivation of the forward model in FP. In the FP system used in our work, the TFT panel illuminates one light spot at a time. After passing through a condenser lens or propagating over a sufficiently long distance, the light emitted from each point source on the TFT panel can be approximated as a plane wave based on the principles of Fraunhofer diffraction. Specifically, the field at the target plane $U_{i}$ is given by:

$$U_{i}\left( x,y \right)= \frac{e^{ikz}e^{\frac{ik(x^{2}+y^{2})}{2z}}}{i\lambda z}\iint_{-\infty}^{\infty} U_{s}\left( \xi,\eta\right)e^{\frac{-i2\pi(x\xi+y\eta)}{\lambda z}}d\xi d\eta$$

Here, $U_{s}\left( \xi,\eta\right)$ represents the optical field at the TFT panel, $U_{i}\left( x,y \right)$ denotes the field at the target plane, $z$ is the propagation distance, $\lambda$ is the wavelength, and $k$ is the wave number. Since only a small light spot is illuminated on the TFT panel at any given time, $U_{s}$​ can be approximated as a delta function. Under this approximation, the resulting field $U_{i}$​ at the target plane corresponds to a plane wave, which can be expressed as:

$$U_{i}=e^{i(k_{x_{n}}x+k_{y_{n}}y)}$$

where the spatial frequencies $k_{x_{n}}$​​ and $k_{y_{n}}$ are given by:

$$k_{x_{n}}=\frac{x_{n}}{z}, k_{y_{n}}=\frac{y_{n}}{z}$$

In this formulation, $x_{n}$​ and $y_{n}$​ represent the coordinates of the illuminated spot on the TFT panel, and $z$ is the propagation distance. For simplicity, constant phase and amplitude terms have been omitted. According to Fourier optics, the image formation process in a coherent FP system can be described as:

$$a_{output}(x,y)={(a}_{input}(x,y)\cdot e^{i\left( k_{x_{n}}x+k_{y_{n}}y \right)})\otimes h(x,y)$$

Where $a_{input}\left( x,y \right)$ denotes the complex field of the sample under normal illumination, $a_{output}\left( x,y \right)$ is the field at the image plane under oblique illumination from the TFT panel, and $h(x,y)$ is the coherent point spread function (PSF) of the imaging system. The convolution operation is represented by $\otimes$. Applying the Fourier transform to both sides give:

$$A_{output}\left( k_{x},k_{y} \right)=A_{input}\left( k_{x},k_{y} \right)\cdot H(k_{x}-k_{x_{n}},k_{y}-k_{y_{n}})$$

This formulation represents the forward model of FP microscopy. As evident from the above equation, each distinct oblique illumination angle realized through the TFT panel effectively shifts the sample's Fourier spectrum relative to the system's transfer function. In this context, the transfer function of FP microscopy acts as a filter that selects a specific portion of the sample’s Fourier domain, depending on the illumination angle. To reconstruct a high-resolution image that faithfully captures both amplitude and phase information, it is necessary to acquire a sufficient number of images under varying oblique illumination angles. The details of the reconstruction process are described in the following section.

The FP reconstruction enables the synthesis of high-resolution amplitude and phase images from a series of low-resolution captures. The process unfolds through a series of well-defined steps, beginning with pattern generation, where a TFT liquid crystal array dynamically controls the illumination angles by modulating the transparency of individual pixels. The illumination strategy employs a spiral scanning pattern, initiating from the center and progressively expanding outward, capturing a total of 81 images over approximately 4 seconds. The TFT array allows precise activation of specific angled illumination, eliminating the need for mechanical scanning and enhancing system efficiency. This angle-varying illumination ensures comprehensive sampling of the sample’s Fourier space, which is critical for reconstructing high-fidelity images of samples.

The reconstruction process then transitions to an iterative algorithm to recover the high-resolution complex field, encompassing both amplitude and phase information. The algorithm initiates with a high-resolution image guess, expressed as $\sqrt{I_{h}}e^{i\phi_{h}}$, where $I_{h}$ represents the intensity and $\phi_{h}$ the phase. The Fourier transform of this guess is computed, and a sub-region of the Fourier spectrum, corresponding to the spatial frequency support of a single low-resolution image, is selected and subjected to a low-pass filter tailored to the metalens’s NA. An inverse Fourier transform generates a low-resolution image $\sqrt{I_{l}}e^{i\phi_{1}}$, which the amplitude is updated from the square of measured intensity corresponding to the illumination angle. The updated image’s FT, $\sqrt{I_{lm}}e^{i\phi_{1}}$, is computed, and this process is repeated iteratively for different incident angles, as dictated by the spiral illumination sequence. The overlapping Fourier regions are stitched together by directly replacing the corresponding regions in the reconstructed Fourier space with the newly measured data, ensuring consistency with the measured intensities. Once the Fourier spectrum is refined, an inverse FT transforms the recovered data back to the spatial domain, yielding the final high-resolution image.

Section 3: Deep learning architecture and training details


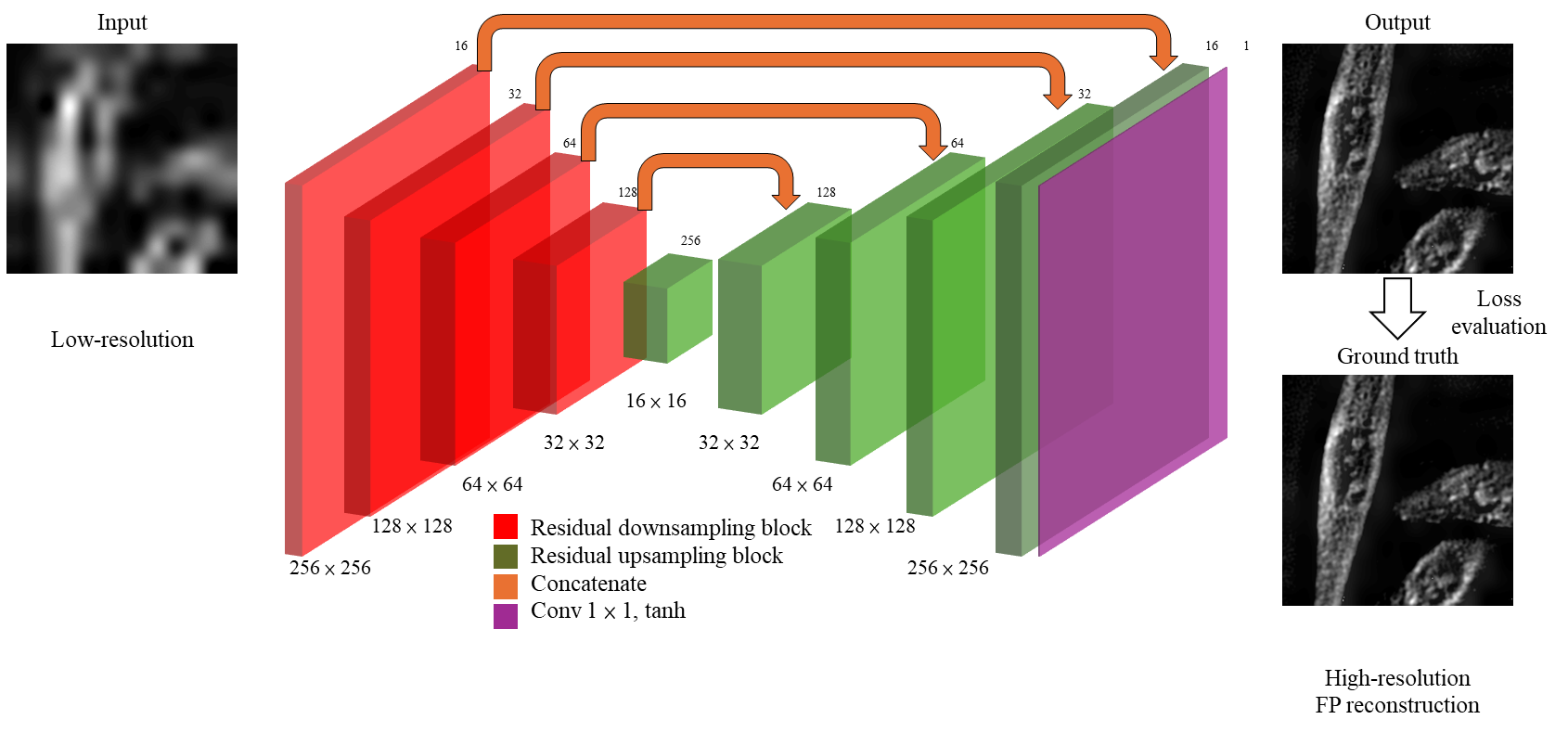


Figure S3. RCNN architecture for FP resolution enhancement. The model receives a low-resolution intensity image as input and outputs a high-resolution prediction, trained to match the FP-reconstructed ground truth. The architecture follows a U-net-inspired encoder–decoder design composed of four residual downsampling blocks and four residual upsampling blocks, with skip connections bridging corresponding layers. A final 1×1 convolutional layer with tanh activation generates the enhanced image. Training is performed using MAE as the loss function by comparing the output with the high-resolution FP reconstruction.

Section 4: Dataset for model training


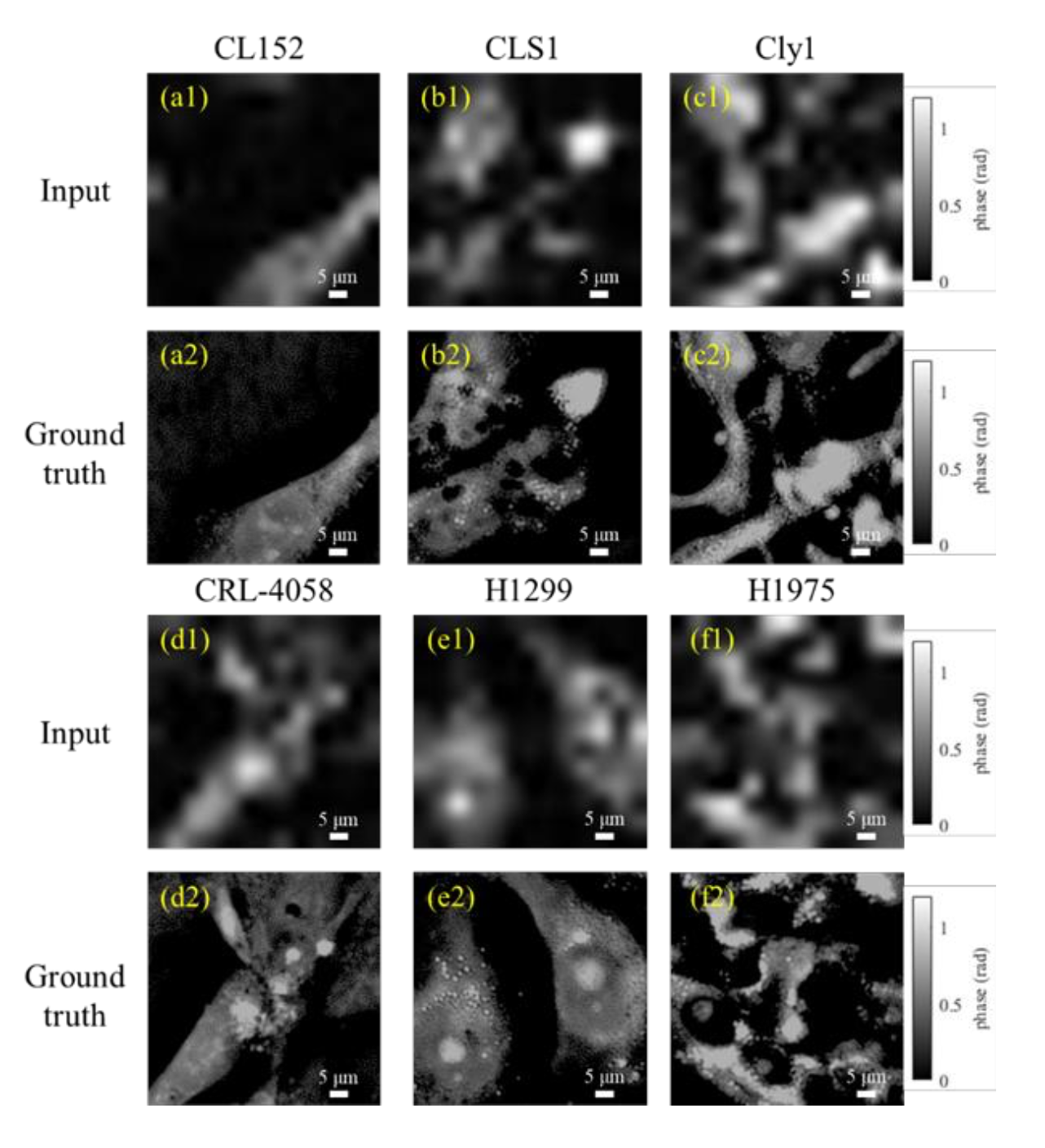


Figure S4. Training dataset used for the ResNet.

The first row (a1-f1) shows the raw low-resolution intensity images of CL152, CLS1, Cly1, CRL-4058, H1299, and H1975 acquired directly from the FP microscope under single-angle illumination. These serve as the input images for model training. The second row (a2–f2) displays the corresponding high-resolution images reconstructed using standard FP algorithms, which are used as ground truth labels to supervise the network. This dataset highlights the diversity in cell morphology across different types and supports the generalizability of the trained model.


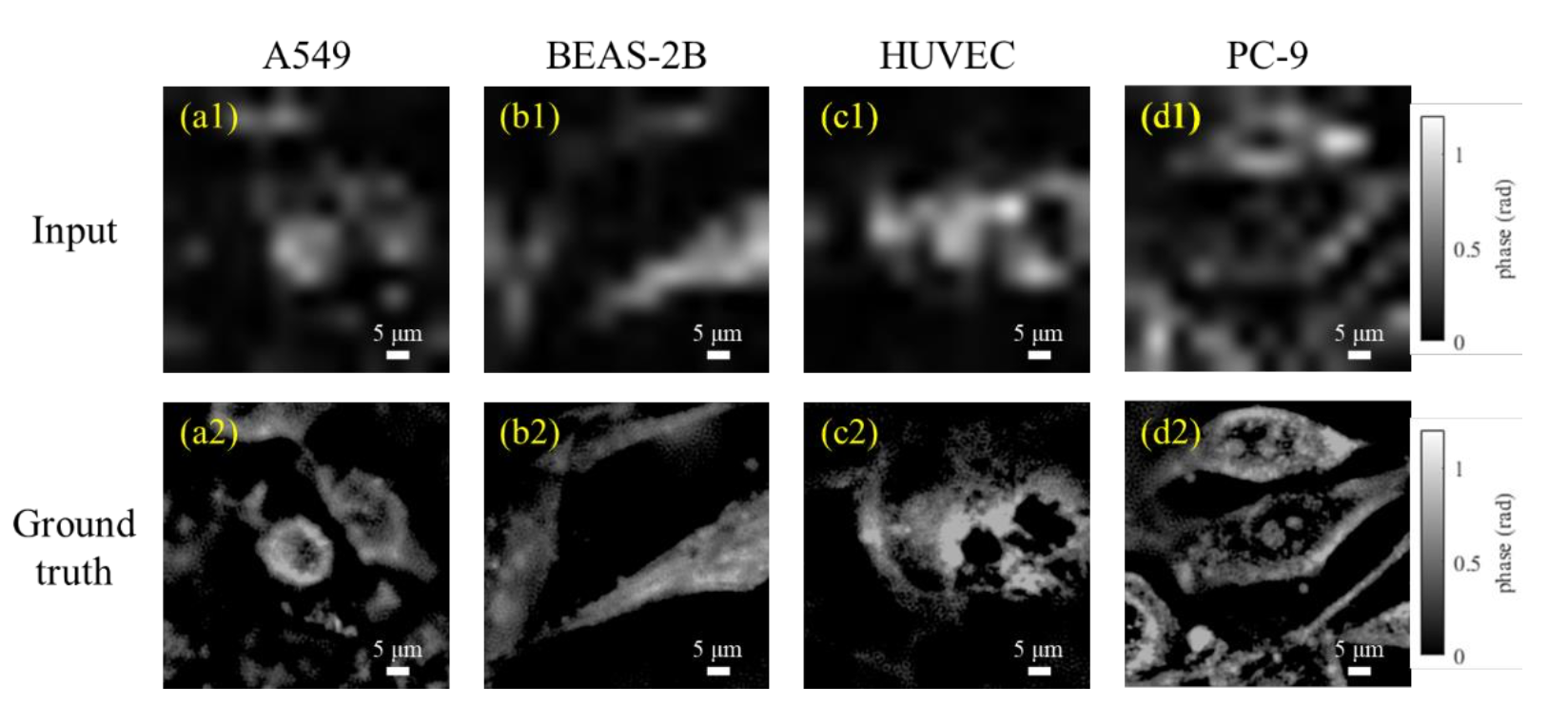

Figure S5. Validation datasets used for the ResNet.

To assess the model’s generalizability beyond the training data, a separate validation dataset comprising previously unseen cell images is employed. This step is critical to evaluate the network’s ability to reconstruct high-resolution phase features in real-world scenarios and to monitor potential overfitting. Each column (a-d) corresponds to a distinct biological cell type, A549, BEAS-2B, HUVEC, and PC-9. The first row (a1-d1) shows the low-resolution input images acquired under single-angle illumination. The second row (a2–d2) presents the corresponding high-resolution reconstructions obtained via conventional FP algorithms, serving as the ground truth for validating the model’s output. This dataset ensures that the trained RCNN model maintains robust reconstruction quality across a variety of cell types not encountered during training.

Section 5: Dry mass

Section 6: Metalens simulation

Figure S6. Electric field intensity profiles of metalens using FDTD simulation at different wavelengths

The performance of metalens was evaluated by using finite-difference time-domain (FDTD) simulations. Due to computational resource limitations, a scaled-down metalens model was used, maintaining the same numerical aperture (NA = 0.05) as the experimental design. The simulated model was reduced to a diameter of 20 µm. The metalens was designed for a central wavelength of 532 nm, where the simulated focusing efficiency reaches approximately 80%, forming a well-defined focal spot near the designed focal plane. Figure S6 shows the simulated electric field intensity distributions (x-z plane) at illumination wavelengths of 450 nm, 532 nm, and 633 nm. At non-design wavelengths, the focal spot becomes axially shifted due to chromatic dispersion, reflecting the wavelength-dependent phase shift of the unit cells.

Section 7: Metalens fabrication


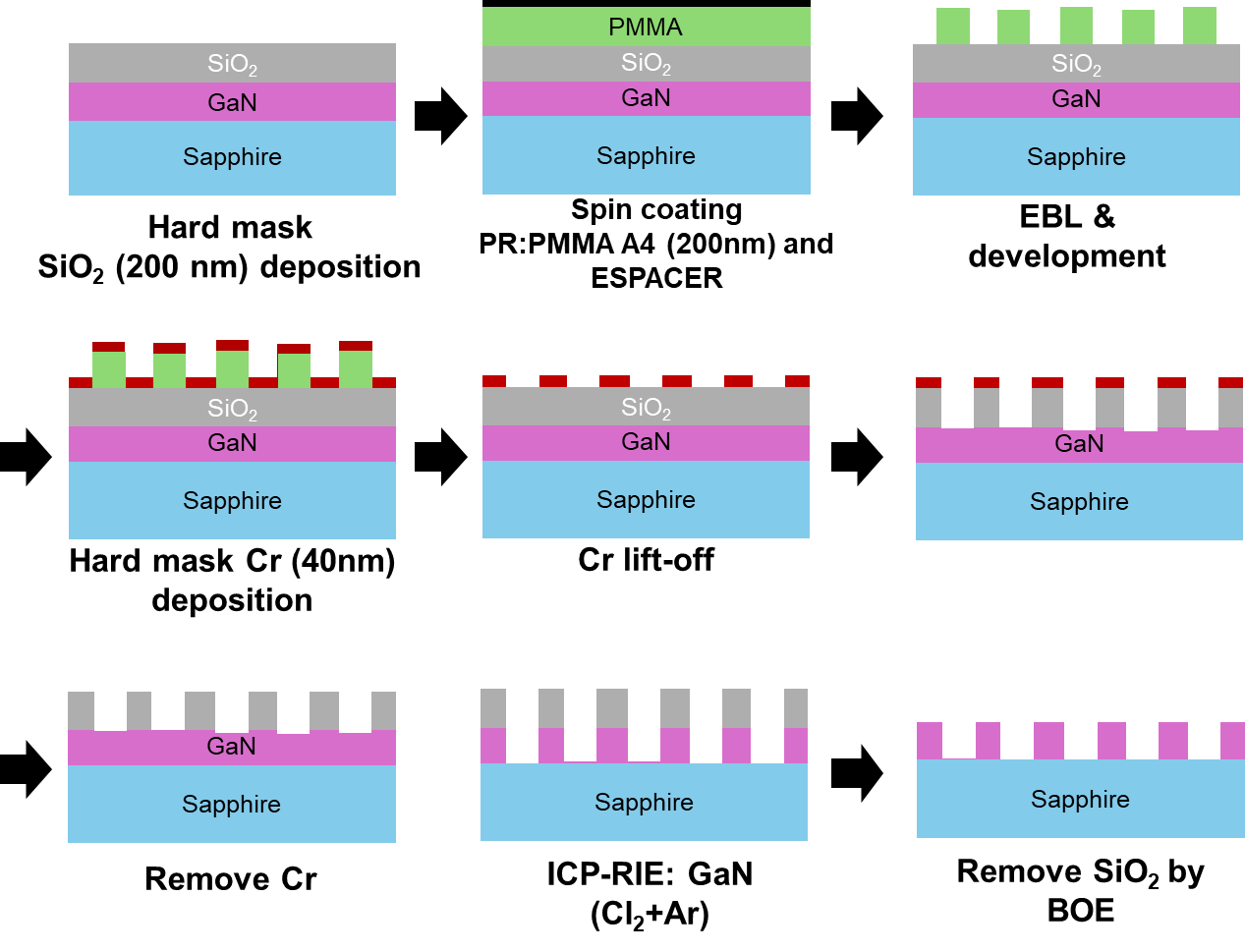


Figure S7. Electron beam lithography for metalens fabrication
